# Modulation of Innate Immunity by lignin-Carbohydrate, a Novel TLR4 Ligand, Results in Augmentation of Mucosal IgA and Systemic IgG Production

**DOI:** 10.3390/ijms19010064

**Published:** 2017-12-26

**Authors:** Ryohei Tsuji, Kumiko Ikado, Daisuke Fujiwara

**Affiliations:** Central Laboratories For Key Technologies, Kirin Co., Ltd., Fukuura 1-13-5, Kanazawa-ku, Yokohama 236-0004, Japan; Kumiko_Ikado@kirin.co.jp (K.I.); d-fujiwara@kirin.co.jp (D.F.)

**Keywords:** lignin-carbohydrate, innate immunity, dendritic cell, Toll-like receptor 4 (TLR4), mucosal immunology, immunoglobulin A (IgA), vaccine adjuvant

## Abstract

Previous study revealed that a specific lignin-carbohydrate preparation, named as lignin-rich enzyme lignin (LREL) derived from plant husk, is a novel toll-like receptor 4 ligand and shows a potent immune-stimulatory activity against dendritic cells (DCs) in vitro. In this report, we investigated immune-stimulatory activity of LREL in vivo. Single intraperitoneal (i.p.) or oral treatment of LREL elicited activation of systemic and mucosal DCs, which were accompanied by significant elevation of cell surface activation markers and ratio of IL-12p40 producing cells. In addition, LREL-fed mice showed not only mucosal DCs activation but also significant increase of IFN-γ^+^ CD4^+^ T cells in mesenteric lymph node (MLN), respectively. We further examined the effect of LREL oral immunization in combination with ovalbumin (OVA) on the activation of acquired immune system. In LREL administered group, total mucosal IgA concentration was significantly increased, while antigen-specific immunoglobulin A (IgA) concentration was not changed between groups. On the other hand, both total and antigen-specific IgG concentrations in plasma were significantly increased in the LREL administered group. Taken together, oral treatment of LREL is able to affect mucosal and systemic antibodies induction and might be useful for effective immune-stimulatory functional foods and mucosal vaccine adjuvant.

## 1. Introduction

Lignin is a well-known major natural compound that is derived from the cell walls of plants [1]. It has been reported that lignin macromolecules are formed by random dehydrogenative polymerization of three major phenylpropane units [2]. Some polysaccharides in the cell walls are linked to lignin through hydroxycinnamic acids, forming ester-ether bridges which form structurally complex lignin-carbohydrates [3,4,5]. Due to the molecular complexity, stable preparations of lignin-carbohydrates have proved to be difficult, and very little attention has been paid to the biological activities of lignin-containing substances. We previously found that lignin-rich enzyme lignin (LREL), one of the fractions extracted from edible plant tissues which contains lignin-carbohydrate, was able to activate bone marrow derived dendritic cells (BM-DCs) and its activity was comparable to LPS [6]. In addition, LREL derived from barley husk was shown to activate BM-DCs via toll-like receptor 4 (TLR4) [6].

Antigen presenting cells (APCs), which are mainly composed of dendritic cells (DCs) and macrophages, are particularly important immune cells for linking innate and adaptive immune systems. APCs uptake pathogens by phagocytosis and present pathogen-derived antigens to naïve T cells. It is well-proven that phagocytosis mediated antigen presentation, accompanied by high expression of co-stimulatory molecules and inflammatory cytokines, is involved in the development of antigen-specific acquired immunity [7]. A typical vaccine contains antigens derived from inactivated or attenuated forms of various pathogens and adjuvants, to activate acquired immunity against particular diseases.

Mucosal vaccines are advantageous compared with systemic vaccines from the contexts of production and regulation [8,9]. Mucosal vaccines are practical for mass vaccination because of their ease of administration, improved compliance and the possibility of delivery without personal medical training, and do not involve risks of spreading blood-derived infections which may occur with contaminated needle injection [10,11]. Oral vaccines represent the biggest challenge for mucosal vaccine development because of the harsh gut environment, which degrades antigenic epitopes, and mucosal tolerance, which protects against unwanted immune responses to digested antigens. Vaccine adjuvants play an important role in eliciting a sufficient antigen-specific immune response, including antibody production [12].

Toll-like receptors (TLRs), which consist of a family of pattern-recognition receptors, are widely expressed on both immune cells, including DCs, macrophages and natural killer (NK) cells, and non-immune cells, such as fibroblast, epithelial cells and keratinocytes [13]. They recognize specific molecular patterns in pathogenic microbial components and in some endogenous ligands, and act as the first defense line against many bacteria and play an important role in the innate immune system [14,15]. Due to their strong immune-modulatory functions, many TLR ligands are expected to be vaccine adjuvants, which are able to directly activate APCs, and, in fact, some of them have proven effects as vaccine adjuvants in clinical trials [12,16].

In this report, we focused on the in vivo immune-modulatory effects of LREL, which was originally discovered as a novel TLR4 ligand (TLR4L) derived from barley husk, and showed that it activated splenic (SPN) DCs and NK cells after single dose administration via a systemic route. We also showed that oral administration of LREL activated the mucosal immune systems, promoted total IgA production in mucosa, and increased both total and antigen-specific IgG production in plasma. These data suggested that LREL might be useful for plant derived-anti infectious agents or oral vaccine adjuvants.

## 2. Results

### 2.1. Systemic Dendritic and NK Cell Activation by Intraperitoneal Treatment

**Figure 1:**
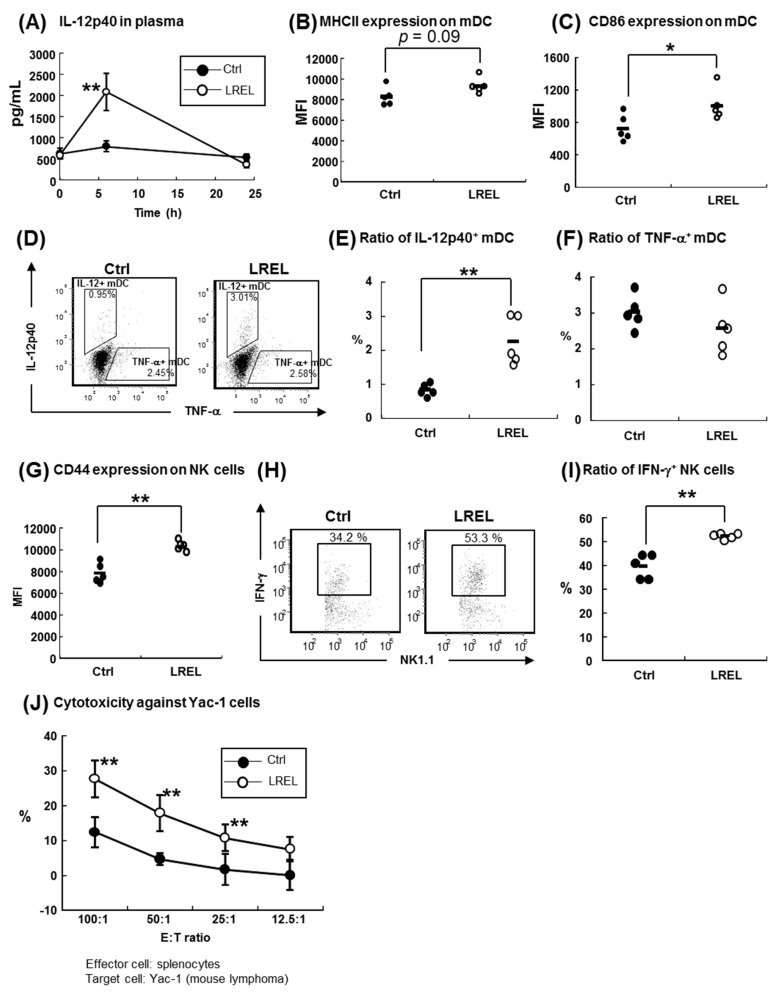
Splenic dendritic and NK cell activation by LREL i.p. treatment. Healthy C57BL/6J mice were divided into control or LREL groups (*n* = 5 in each group). Mice in the LREL group were intraperitoneally administered 200 μg of LREL dissolved in phosphate buffer saline (PBS). Mice in the control group were administered the same volume of PBS. All mice were sacrificed 24 h after treatment. (**A**) Sequential IL-12p40 concentrations in plasma. Data are shown as mean ± SD (*n* = 5). (**B**,**C**) MHC class II and CD86 expression levels in SPN mDCs, respectively. The expression levels of cell surface activation markers were analyzed by flow cytometry and evaluated as median fluorescence intensity (MFI). mDCs were defined as “CD11c^+^ CD11b^+^ CD8α^−^ PDCA-1^−^” in the low density cells of splenocytes. (**D**) Representative ratio of IL-12p40 and TNF-α producing mDCs from the control and LREL groups. (**E**,**F**) Ratio of IL-12p40 and TNF-α producing mDCs, respectively. The ratio of IL-12p40 producing mDCs was significantly increased in the LREL group, while that of TNF-α producing mDCs was not changed between the two groups. (**G**) CD44 expression levels on splenic NK cells were analyzed by flow cytometry and evaluated as MFI. NK cells were defined as “NK1.1^+^ CD3ε^−^” in total splenocytes. (**H**) Representative IFN-γ producing NK cells from the control and LREL group. (**I**) Ratio of IFN-γ producing NK cells. The short line in (**B**,**C**,**E**–**G**,**I**) represent the mean value (*n* = 5). (**J**) Cytotoxicity of NK cells against Yac-1 cells. Total splenocytes and calcein-AM labeled Yac-1 cells were co-cultured in various E:T ratios. After 4 h of co-culturing, ratios of Yac-1 specific lysis were calculated. Data are shown as mean ± SD (*n* = 5). ** *p* < 0.01, * *p* < 0.05 compared to control (Student’s *t* test). These data are representative of two independent experiments.

We previously reported that LREL strongly activated DCs, in vitro, via TLR4 [6], however, it was not determined whether LREL could activate innate immunity in vivo. To determine the activity of LREL, C57BL6/J mice were intraperitoneally (i.p.) injected with 200 μg of LREL; the concentration of IL-12p40 in plasma samples was transiently upregulated at 6 h after injection (Figure 1A). Furthermore, the activities of SPN DCs were evaluated using flow cytometry (Figure S1A). As shown in Figure 1B–F, the expression of CD86 on mDCs (defined as CD11c^+^ CD11b^+^ CD8α^−^ PDCA-1-) and the ratio of IL-12 producing mDCs were significantly increased, and the expression level of MHC class II on mDCs tended to be increased (*p* = 0.09) compared to the control group, while TNF-α producing mDCs were not affected. In addition, we analyzed the activities of pDC (defined as CD11c^int^ PDCA-1^+^) and CD8^+^ DC (defined as CD11c^+^ CD11b^−^ CD8α^+^ PDCA-1^−^). As a result, the expression levels of CD86 on pDC were significantly increased (*p* < 0.01), while those of MHC class II on pDC were not changed between two groups (*p* = 0.14) (Figure S2A). Furthermore, the expression levels of CD86 and MHC class II on CD8^+^ DC were not significantly changed, while the mean values of these expression in LREL group were higher than those in the Ctrl group (MHC class II: *p* = 0.18, CD86: *p* = 0.13) (Figure S2B). These data suggested that LREL could stimulate SPN DCs in vivo by intraperitoneal (i.p.) treatment and mDCs were the most sensitive to LREL stimulation. Therefore, we focused on the mDC activities in further experiments. Since NK cells are known to be activated by the TLR4 ligand both directly [17] and indirectly [18], we also evaluated NK cell activities (Figure S1B). As shown in Figure 1G, the expression level of CD44, an activation marker which triggers cytotoxicity in NK cells [19,20], was significantly increased on NK cells (defined as NK1.1^+^ CD3ε^−^) in LREL-treated mice, compared with control mice. In addition, the ratio of IFN-γ producing NK cells was also significantly increased (Figure 1H,I). Furthermore, the cytotoxic activity of NK cells against Yac-1 cells, representative cells to be sensitive to NK cells [21], was increased in the LREL treated group (Figure 1J). These data suggested that LREL i.p. treatment activated splenic NK cells as well as splenic mDCs. Taken together, LREL could activate innate immune system both in vitro [6] and in vivo via the i.p. route.

### 2.2. Resistance against Heat and Acid Treatment of LREL

**Figure 2:**
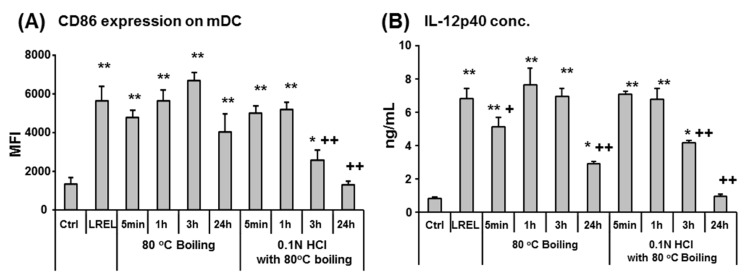
Effect of heat and acid treatment on LREL activity. LREL were boiled at 80 °C with or without 0.1 N HCl for different time periods and then neutralized with 0.1 N NaOH to evaluate heat and acid resistance of LREL. Flt-3L-induced BM-DCs were then incubated with heat and acid treated LREL at a concentration of 1 μg/mL. Untreated LREL were used as a positive control. After 24 h, cells were analyzed by flow cytometry (gated on CD11b^+^ CD11c^+^) to determine the cell surface expression levels of CD86. Cell supernatants were analyzed by ELISA to determine the concentration of IL-12p40. (**A**) Expression level of CD86 (expressed as MFI) on the cell surface of DCs stimulated with 1 μg/mL LREL untreated or treated with indicated conditions. Data are shown as mean ± SD (*n* = 3). (**B**) Culture supernatants were assayed for IL-12p40, as a representative inflammatory cytokine, by ELISA. Data are shown as mean ± SD (*n* = 3). Immune-stimulatory activity of LREL tended to be attenuated by more than 24 h boiling and became weaker as the length of acid treatment time increased. * *p* < 0.05; ** *p* < 0.01 compared with control (Ctrl). + *p* < 0.05; ++ *p* < 0.01 compared with untreated LREL (LREL) (one-way ANOVA with Bonferroni post-hoc test). These data are representative of two independent experiments.

Since LREL is derived from an edible plant, it could be used as immune-stimulatory food material; this would require stable physiological activity so that it would not be degraded via the oral route of administration. To investigate physiological stability, we focused on resistance against heat and acid treatment. As shown in Figure 2, both CD86 expression and IL-12p40 production were not attenuated by 3 h boiling treatment. 0.1N HCl acidification decreased the immune-stimulatory activity, although its activity was sustained for 3 h treatment. The gastric residence time of dietary fibers is known to be 2 to 3 h, suggesting that the immune-stimulatory activity of LREL was resistant enough in the dietary tract for oral administration.

### 2.3. Activation of Mucosal Immunity by Oral Administration

**Figure 3:**
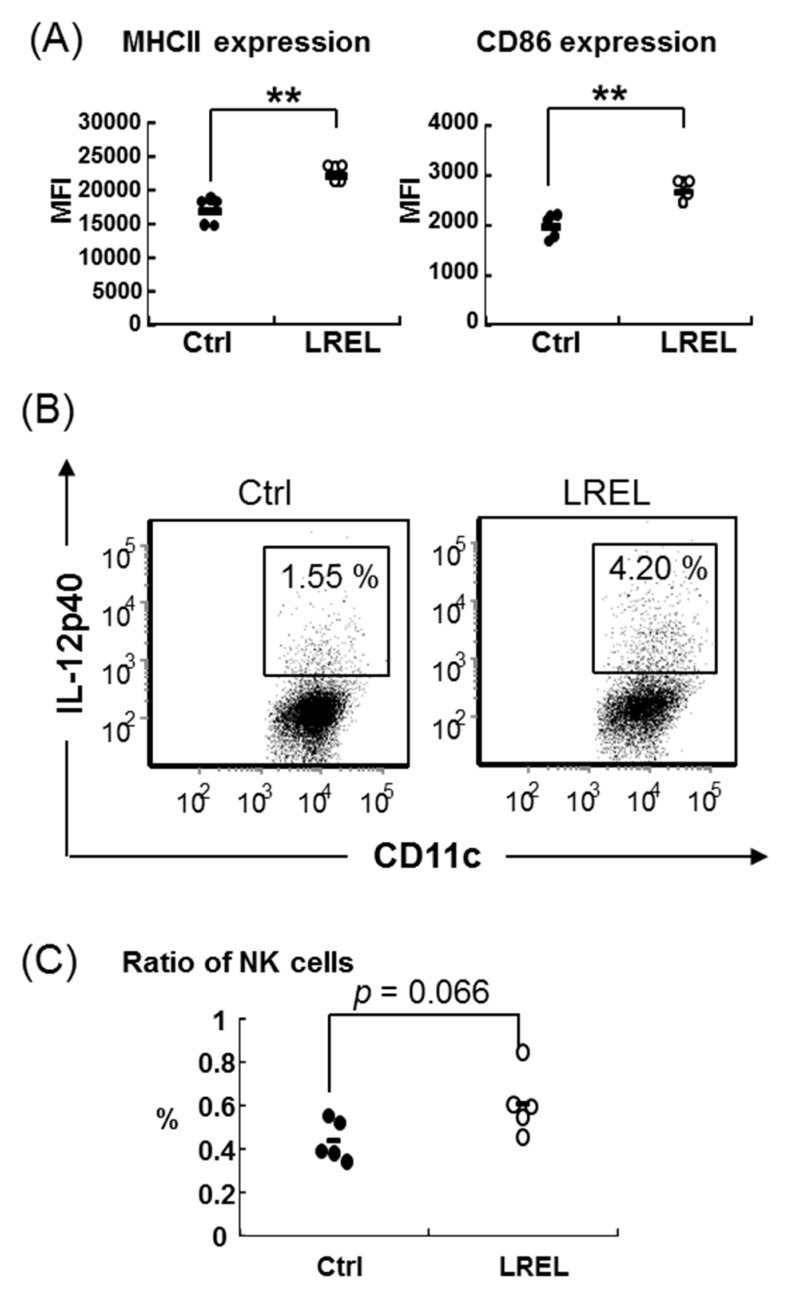
Mucosal DCs activation and NK cells increase by LREL single dose oral treatment. Healthy C57BL/6J mice were divided into control and LREL groups (*n* = 5 in each group); mice in the LREL group received oral administration of 10 mg LREL dissolved in PBS and mice in the control group received oral administration of the same volume of PBS. After 24 h, all mice were sacrificed and the activities of mDCs (gated on CD11c^+^ CD11b^+^ CD8α^−^ PDCA-1^−^) and NK cells (gated on NK1.1+ CD3ε^−^) in MLN were analyzed by flow cytometry. (**A**) Comparison of cell surface activation marker expression on MLN mDCs between the control and LREL group. Expression levels of MHC class II (left panel) and CD86 (right panel) were analyzed by flow cytometry (expressed as MFI). The short line represents the mean value (*n* = 5). MLN mDCs were significantly activated by LREL oral treatment. (**B**) Representative ratio of IL-12p40 producing mDCs from the control and LREL group. Low density cells of MLN in the same group were pooled and analyzed by FACS. (**C**) Comparison of the ratio of NK cells in MLN. The ratio of NK cells was moderately increased in LREL group. ** *p* < 0.01 compared to control (Student’s *t* test).

**Figure 4:**
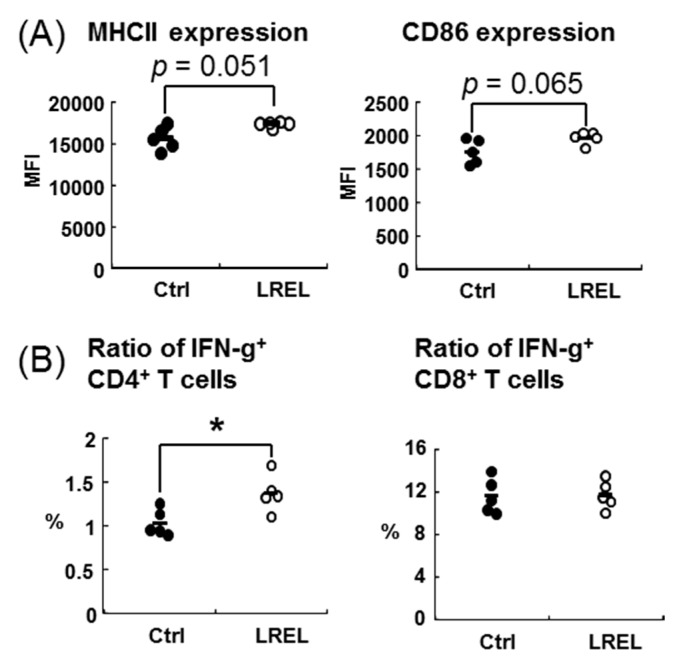
Activation of mucosal acquired immune system by LREL oral treatment for two weeks. Healthy C57BL/6J mice were divided into control and LREL groups (*n* = 5 in each group); mice in the LREL group received oral administration AIN93G containing 10 mg/day of LREL and mice in the control group received oral administration of AIN93G without LREL for two weeks. (**A**) Comparison of cell surface activation marker expression on MLN mDCs between the control and LREL group. Expression levels of MHC class II (left panel) and CD86 (right panel) were analyzed by flow cytometry (expressed as MFI) and mDCs were defined as “CD11c^+^ CD11b^+^ PDCA-1^−^” in low density cells. MLN mDCs tended to be activated by LREL oral treatment for 2 weeks. (**B**) Ratios of IFN-γ producing T cells in MLN were analyzed by flow cytometry. T cells were defined as “CD3ε^+^ NK1.1^−^“. Ratio of IFN-γ producing CD4^+^ T cells (left panel) was significantly increased in the LREL group, while that of CD8^+^ T cells (right panel) was not changed. The short line in all figures represents the mean value (*n* = 5). *, *p* < 0.05 compared to control (Student’s *t* test). These data are representative of two independent experiments.

To determine the effect of LREL on mucosal innate immune system, C57BL6/J mice received oral administration of a single dose of 10 mg LREL. After 24 h, the expression levels of MHC class II and CD86 on mDCs in mesenteric lymph node (MLN) in the LREL group were significantly up-regulated (Figure 3A) and the ratio of IL-12 producing DCs was increased compared with the control group (Figure 3B). In addition, the ratio of NK cells were moderately increased (*p* = 0.066) (Figure 3C), while the expression levels of CD44 on NK cells and the ratio of IFN-γ^+^ NK cells were not changed between two groups (Figure S3). Furthermore, we also evaluated the SPN innate immune cells, however, there was no significant difference in activities of both DCs and NK cells (data not shown). These data suggested that oral administration of LREL could stimulate mucosal innate immunity. We then determined the effect on mucosal acquired immunity using 10 mg/day of LREL oral administration for two weeks. AIN93G rodent diet was used as basal diet in this study, and for the LREL group, 10 mg/day of LREL was mixed in it. There was no significant difference in body weights between Ctrl and LREL group. As shown in Figure 4A, the expression levels of MHC class II and CD86 on MLN mDC in LREL-fed group tended to be increased (MHC class II: *p* = 0.051, CD86: *p* = 0.065) compared to the control group. The ratio of IFN-γ^+^ CD4^+^ T cells in MLN was significantly increased compared to the control, while that of IFN-γ^+^ CD8^+^ T cells was not changed (Figure 4B). These data suggested that oral administration of LREL could induce activation of not only mucosal innate immune systems by single dose treatment but also acquired immune systems by two weeks oral treatment.

### 2.4. Increase in Antibody Production in Intestinal Contents and Plasma after Oral Immunization

**Figure 5:**
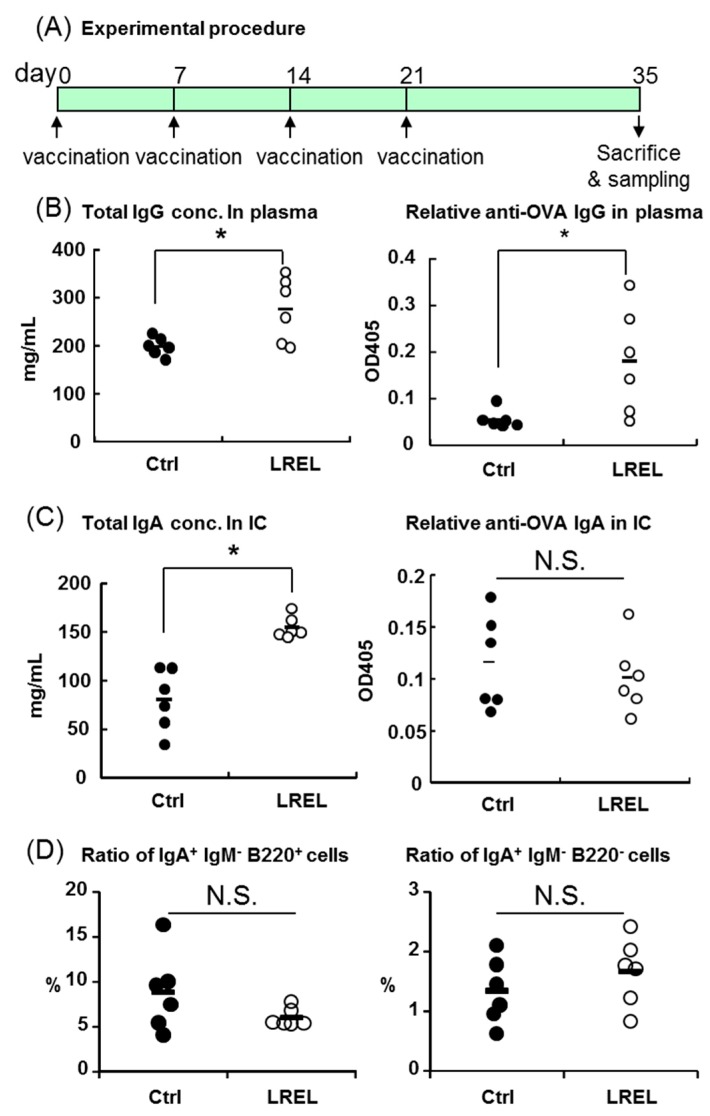
Systemic IgG and mucosal IgA induction by oral LREL stimulation in combination with OVA. (**A**) Experimental procedure of LREL and OVA vaccination. Healthy BALB/C mice were divided into the control and LREL groups (*n* = 6) and vaccinated with 1 mg of OVA with/without 1 mg LREL once a week (day 0, 7, 14 and 21), and all mice were sacrificed two weeks after the final vaccination (day 35). (**B**) Total IgG concentration in plasma (left panel) and relative concentration of anti-OVA IgG in plasma (right panel). Total IgG and anti-OVA IgG in plasma were significantly increased in the LREL group. (**C**) Total IgA concentration in the intestinal contents (left panel) and relative concentration of anti-OVA IgA in the intestinal contents (right panel). Total IgA concentration in the intestinal contents was significantly increased in the LREL group, while anti-OVA IgA concentration was not changed between the control and LREL group. (**D**) Comparison of the ratio of IgA^+^ B cells in Peyer’s patches. Both the ratio of IgA^+^ IgM^−^ B220^+^ cells (left panel) and IgA^+^ IgM^−^ B220^−^ cells (right panel) was not significantly changed between two groups. The short line in (**B**–**D**) represents the mean value (*n* = 6). * *p* < 0.05 compared to the control (Student’s *t* test). These data are representative of two independent experiments.

Since we showed that LREL could activate mucosal CD4^+^ T cells but not CD8^+^ T cells by oral treatment, we hypothesized LREL might induce antibodies production in vivo. To determine the effect of LREL, concentrations of antigen-specific and total antibodies were measured after four times oral administration of antigen with LREL. Prior to the experiment, a dose-finding experiment was performed and found that mucosal DCs could be activated by 1 mg LREL administration as well as 10 mg (data not shown). Therefore, dose of LREL immunization was set at 1 mg/head. As shown in Figure 5A, mice were orally immunized once a week for three weeks with 1 mg OVA in the presence or absence of 1 mg LREL to healthy BALB/c mice, and the concentrations of total and OVA-specific antibodies in plasma and intestinal contents (ICs) were measured two weeks after the final immunization. As a result, both total IgG and anti-OVA IgG concentrations in plasma were significantly increased in the LREL group compared with the control (Figure 5B). Total IgA concentration in the ICs was significantly increased in LREL group, while anti-OVA IgA concentration was not changed (Figure 5C). On the other hand, there was no difference in the ratios of IgA^+^ IgM^−^ B220^+^ cells and IgA^+^ IgM^−^ B220^+^ cells in Peyer’s Patches (PPs) between two groups (Figure 5D). These data suggested that oral immunization of LREL could lead to the induction of mucosal total antibody production and systemic total and antigen-specific antibody productions.

## 3. Discussion

We previously reported that LREL derived from edible plants could strongly activate BM-DCs via TLR4 and barley husk-derived LREL had the strongest activity as a source [6]. The present study was conducted to elucidate the effects of barley husk-derived LREL in vivo via i.p. and oral administration routes. A single dose treatment of LREL stimulated the innate immune system via both the i.p. and the oral route. In addition, long term oral administration of LREL activated acquired immunity and the continuous administration of LREL led to an increase in mucosal and systemic antibody production.

When LREL was administrated intraperitoneally, plasma IL-12p40 concentration was transiently increased, and IL-12p40 producing mDC in SPN was significantly increased. On the other hand, surprisingly, TNF-α producing mDC in SPN was not changed between two groups. In our previous study, TNF-α was induced from BM-DCs by LREL stimulation [6]. Since the same lot of LREL was used in in vitro study [6] and in vivo study, it was possible that the reactivity against LREL was different between Flt-3L induced BM-DCs in vitro and SPN DCs in vivo due to the difference of DC source. In addition to DC activation, NK cells were significantly activated; indicators of their activation such as the expression level of CD44 on cell surface, the ratio of IFN-γ producing cells and the cytotoxic activities against Yac-1 cells. IFN-γ is considered to be one of the most prominent cytokines produced by NK cells [22,23]. In previous reports, TLR4 was expressed on the surface of NK cells [17], and NK cells could be activated via IL-12 and IL-15 stimulation, which were produced by DC activation [24,25]. LREL might, therefore, possess anti-viral and anti-tumor activities through NK cell activation, using both direct and indirect mechanisms.

We also revealed that the immune-stimulatory activity of LREL was resistant to heat and acid treatment, suggesting stability under high temperature and low pH conditions. Generally speaking, lignin-carbohydrates are well-known to be resistant to digestive enzyme treatment [26]. Therefore, we hypothesized that the immune-stimulatory activity of LREL could be sustained in the digestive tract and may stimulate gastro-intestinal immunity via oral administration. In fact, the single dose study of LREL oral administration showed a significant increase of MHC class II and CD86 expression of mDCs, the ratio of IL-12p40 producing mDCs and the ratio of NK cells in MLN. DCs were well-known innate immune cells that linked between innate immunity and acquired immunity [7]. In addition, a previous report showed that NK cells were recruited in matured DC-draining lymph node and they induced acquired immunity [27]. Therefore, it was suggested that LREL oral administration might activate acquired immune cells including vaccination via activation of DCs and the recruitment of NK cells. The longer term, daily, oral administration of LREL demonstrated activation of mucosal innate immunity, from the elevation of expression of activation markers of DCs, and acquired immunity from the significant increase of IFN-γ producing CD4^+^ T cells in MLN. These data suggested that LREL was a potent stimulator of acquired immunity as well as innate immunity. Compared to the single dose study, mDC activation in long-term administration study was moderate. Since mucosal DCs were gradually and continuously stimulated in long-term administration study, it is suggested that the activity of mDC was regulated by negative feedback due to homeostasis.

In the OVA and LREL co-administration experiments, both total and anti-OVA IgG in plasma and total IgA in the ICs were significantly up-regulated compared with the control group, while only antigen-specific IgA in the ICs was not increased (Figure 5C). In a preliminary experiment, it was confirmed that anti-OVA IgA concentrations in ICs were significantly increased by CpG ODN 1826 in combination with OVA administration (Figure S4), which was shown to induce antigen specific IgA in mucosa in previous study [28]. These data suggested that there were no problem in the method for anti-OVA IgA measurement. Mucosal IgA class-switch recombination (CSR) is reported to be induced by two different mechanisms: T cell-dependent (TD) IgA CSR and T cell-independent (TI) IgA CSR [29]. TD IgA CSR is predominantly induced at PPs [30], while TI IgA CSR occurs in lamina propria (LP) [31] and isolated lymphoid follicles [32]. Since there was no difference in the ratio of IgA class switched cells (IgA^+^ IgM^−^ B220^+^ cells and IgA^+^ IgM^−^ B220^−^ cells) in PPs between two groups, it is suggested that TI IgA CSR was mainly induced rather than TD IgA CSR by LREL oral immunization. A previous report showed that LP B-1 B cells, which also participate in mucosal TI IgA production [33], were activated by oral administration of LPS [34]. In addition, TLR4 stimulated intestinal epithelial cells induced IgA class switch related genes, such as APRIL and BAFF, and recruited B cells to LP [35].

Orally administered antigens are uptaken by mucosal DCs in mainly two ways; one is to be delivered by microfold (M) cells across the epithelial barrier and uptaken by subepithelial DCs in PPs [36], and the other is to be uptaken directly by LP DCs, which migrate into epithelial layers and extend dendrites into lumens [37,38]. After antigen phagocytosis, DCs move to the nearest draining lymph node (i.e., MLN) and present the antigens to naïve T cells [39,40]. As shown in Figure 3 and Figure 4, MLN DCs were stimulated by oral administration of LREL, suggesting that systemic humoral immunity could be induced via MLN DCs activation and both total and antigen-specific IgG were significantly up-regulated compared to the control group.

In conclusion, we could show the possibility that LREL can be utilized as novel immune-stimulatory functional foods. When LREL was used as functional foods, its extraction method seems to be complicated. In our previous report, it was proved that cellulase treatment is the key treatment for its immune-stimulatory activity. Therefore, we are developing to modify the extraction method by focusing on cellulase treatment. Furthermore, we could report the primitive data for the possibility of mucosal vaccine adjuvant. As previously reported, it is suggested that a human vaccine adjuvant should fulfill stringent requirements in the aspects of efficacy, safety and stability [41]. Our findings indicated that LREL might fulfill minimum essential requirements. Therefore, we suggest that LREL is a potent mucosal vaccine adjuvant that may lead to the novel development of mucosal vaccines, and we are currently exploring this possibility.

## 4. Materials and Methods

### 4.1. Lignin Extraction

Barley husk was derived from KIRIN Brewing Co., Ltd. (Tokyo, Japan). Lignin rich enzyme lignin (LREL) fraction was extracted from the barley husk following a previously published protocol [6]. In brief, the air-dried samples were dewaxed by toluene-ethanol (2:1, *v*/*v*) and then crushed into powders using a multi-bead shocker (Yasui Kikai, Tokyo, Japan). The ground samples were extracted twice with a dioxane-water mixture (90:10, *v*/*v*), followed by another extraction with a dioxane-water mixture (50:50, *v*/*v*). The dioxane-water-extracted residues were washed with water more than once and were treated with a mixture of same volume of *Aspergillus niger* cellulase (Sigma, St. Louis, MO, USA) and *Aspergillus niger* hemicellulase (Sigma) (32 mg/mL of each enzyme) dissolved in 0.2 M sodium acetate buffer (pH 4.8). After filtration through a nylon cloth, the insoluble residues were washed with large amount of water, and LREL fraction was obtained by successive extractions with 90% and 50% dioxane-water.

### 4.2. Mice

Six to ten weeks old female C57BL/6J and BALB/c wild-type mice were purchased from Charles River Laboratories Japan (Kanagawa, Japan). All animal experiments were performed in accordance with the guidelines for care and use of laboratory animals of Kirin Co., Ltd. (Tokyo, Japan). These studies were approved by the Committee for Animal Experiment at Kirin Co., Ltd. The approval IDs were YO09-00158, YO09-00170, YO09-00180 and YO10-00020. The mice were housed, one per cage, in specific pathogen-free conditions under a 12 h light/dark cycle. The temperature in the room was kept at 23 ± 1 °C and 60 ± 15% humidity. Adequate measures were taken to minimize pain and discomfort, taking into account human endpoints for animal suffering and distress.

### 4.3. LREL Intraperitoneal Treatment and Sample Collection

C57BL6/J mice were divided in two groups, the control group and the LREL group, by body weight and the concentration of plasma IL-12p40. Plasma samples for group dividing were collected from the retro-orbital venous plexus three days before grouping. The average of body weights were 16.77 ± 0.62 g in Ctrl group and 16.64 ± 0.38 g in LREL group (*p* = 0.71), respectively. The average of plasma IL-12p40 concentration were 669 ± 140 pg/mL in Ctrl group and 670 ± 87 pg/mL in LREL group (*p* = 0.99), respectively. Mice in the LREL group were injected intraperitoneally with 200 μg of LREL dissolved in PBS and those in the control group were injected intraperitoneally with the same volume of PBS. Plasma samples were collected at 0 h, 6 h and 24 h after injection and were assessed by measuring the concentration of IL-12p40. Twenty four hours after LREL administration, mice were sacrificed and spleens (SPNs) were collected. Splenic lymphocyte activation in response to LREL treatment was examined by FACS analysis. Splenic NK cell activation in response to LREL treatment was examined using a cytotoxicity assay.

### 4.4. Stability of Immune-Stimulatory Activity of LREL against Heat and Acid Treatment

LREL were boiled with or without 0.1 N HCl at 80 °C for 5 min, 1, 3 and 24 h. Upon completion of acidified samples, each sample was neutralized by adding the same volume of 0.1 N NaOH. Neutralized samples were diluted with PBS and used to evaluate immune-stimulatory activities using BM-DCs in vitro.

### 4.5. Induction of BM-DCs and Measurement of Immune-Stimulatory Activity

Flt-3L induced BM-DCs were generated as described previously [6]. After induction of BM-DCs, lignin samples were added at a concentration of 1 µg/mL and cultures were maintained for another 24 h. The immune-stimulatory activities were measured by following the expression levels of cell surface markers using FACS (BD Biosciences, San Jose, CA, USA) and by measuring the concentration of IL-12p40 in the cell culture supernatant using an ELISA (described below). Untreated LREL was used as a positive control.

### 4.6. LREL Oral Treatment and Sample Collection

C57BL6/J mice were divided in two groups, the control group and the LREL group, by body weight and the concentration of plasma IL-12p40. Plasma samples for group dividing were collected and assessed as described above (refer the Section 4.3). In the single dose study, mice in the LREL group were fasted for 12 h before oral administration of 10 mg LREL dissolved in PBS. Mice in the control group were orally administered PBS. Oral administrations of PBS or LREL solution were performed using by feeding needle (Fuchigami, Kyoto, Japan). Twenty four hours after administration, MLNs and SPNs were collected and the activation of lymphocytes in response to LREL was examined by FACS analysis. In the long term oral treatment study, mice in the LREL group were administered AIN93G (Oriental Yeast, Tokyo, Japan) containing 10 mg/day of LREL and those in the control group were administered AIN93G without LREL. After two weeks oral administration, MLNs and SPNs were collected and the activation of lymphocytes in response to LREL was examined by FACS analysis.

### 4.7. Oral Immunization and Sample Collection

Oral immunization to mice was performed as previously reported with some modifications [42]. In brief, BALB/C mice were divided in two groups, the control group and the LREL group, by body weight. Mice were fasted for 12 h and then given 500 μL of 0.1 M sodium bicarbonate solution to neutralize stomach acid before oral immunization. Thirty minutes later, mice were orally immunized with 1 mg of OVA (Seikagaku Corp., Tokyo, Japan) with or without 1 mg of LREL. This oral immunization procedure was conducted on days 0, 7, 14, and 21. Stomach acid neutralization and oral immunization were performed using by feeding needle. Two weeks after the last immunization, samples of intestinal contents (ICs) were obtained by removing 10 cm of small intestine cut near the cecum and passing 4 mL ice chilled PBS containing a protease inhibitor cocktail (BioVision, Milpitas, CA, USA) through the entire intestine. Plasma samples were collected from the retro-orbital venous plexus. PPs were collected and the ratio of IgA^+^ B cells in response to LREL was examined by FACS analysis.

### 4.8. Preparation of DC Fractions

Low density cell fractions from SPN and MLN lymphocytes were prepared as previously reported, with some modifications [43]. Briefly, SPNs or MLNs were minced in Mg^2+^- and Ca^2+^-free HBSS before digestion with 1 mg/mL collagenase IV (Sigma-Aldrich, St. Louis, MO, USA) and 0.2 mg/mL DNase I (Roche, Mannheim, Germany) for 20 min at 37 °C. EDTA was adjusted to 30 mM for the deactivation treatment of enzymes. Erythrocytes from SPN cells were removed by brief exposure to 0.168 M NH_4_Cl at room temperature for 1 min. The cells were resuspended in HBSS. SPN and MLN lymphocytes were filtered through a 70-μm nylon cell strainer (BD) and layered onto 15% Histodenz^TM^ (Sigma-Aldrich) in RPMI 1640 containing 10% FCS (HyClone, South Logan, UT, USA). Cells were separated by centrifugation at 450× *g* for 20 min. Low-density fractions at the interface were collected and washed for FACS analysis.

### 4.9. Antibodies

The following fluorescence conjugated anti-mouse mAbs were purchased from eBioscience (San Diego, CA, USA): CD4-APC (L3T4), CD11c-APC and PE-Cy7 (N418), CD44-PE (IM7), CD86-PE (GL1), MHC Class II-FITC (M5/114.15.2), NK1.1-PE-cy7 (PK136), IgA-FITC (mA-6E1), IgM-APC (II/41) and TNF-α-FITC (MP6-XT22). B220-PerCP and APC-Cy7 (RA3-6B2), CD3ε-PerCP (145-2C11), CD8α-PerCP (53-6.7), CD11b-APC-Cy7 (M1/70), IFN-γ-PE (XMG1.2) and IL-12p40/p70-PE (C15.6) were purchased from BD Pharmingen (San Diego, CA, USA). PDCA-1-APC (JF05-1C2.4.1) was purchased from Miltenyi Biotec (Bergisch Gladbach, Germany).

### 4.10. FACS Analysis

Cells for FACS analysis were stained with fluorescent dye-conjugated Abs (FITC, PE, PerCP, APC, APC-Cy7 and PE-Cy7). After staining, the cells were washed twice with FACS buffer (0.5% BSA in PBS buffer) and suspended in 4% paraformaldehyde for FACS analysis. Data were collected by FACS Canto II (BD Biosciences) and analyzed by FCS Express software (De Novo Software, Glendale, CA, USA). Flt-3L induced BM derived myeloid DCs (mDCs) were defined as CD11c^+^ CD11b^+^ B220^−^, SPN and MLN mDCs were defined as CD11c^+^ CD11b^+^ CD8α^−^ PDCA-1^−^, pDCs were defined as CD11c^int^ PDCA-1^+^, CD8^+^ DCs were defined CD11c^+^ CD11b^−^ CD8α^+^ PDCA-1^−^, NK cells were defined as NK1.1^+^ CD3ε^−^, and T cells were defined as CD3ε^+^ NK1.1^−^, respectively.

### 4.11. Intracellular Cytokine Staining

To stain cytokines produced by DCs, cells in the low-density fraction were harvested and stimulated overnight with Leukocyte Activation Cocktail (BD Biosciences). Cells were resuspended in FACS buffer and stained with anti-CD11c, anti-CD11b and anti-PDCA-1. Cells were then fixed with Cytofix/Cytoperm (BD Biosciences) and then stained with anti-IL-12p40 and anti-TNF-α. For T cell and NK cell cytokine staining, total lymphocytes were stimulated with Leukocyte Activation Cocktail for 4 h. Cells were surface stained with anti-CD3ε, anti-CD4, anti-CD8α and anti-NK1.1 and then fixed with Cytofix/Cytoperm before staining with anti-IFN-γ.

### 4.12. Cytotoxicity Assay

The natural killing activity of SPN cells was determined using the previously reported calcein AM (Molecular Probes, Eugene, OR, USA) releasing assay, with some modification [44]. Briefly, Yac-1 cells (RIKEN BioResource Center, Tsukuba, Japan) were used as the target cells of NK cells. Yac-1 cells were resuspended in RPMI1640 medium supplemented with 10% FCS at a final concentration of 1 × 10^6^ cells/mL and incubated with 15 μM calcein-AM for 30 min at 37 °C with occasional shaking. Experiments were performed in round bottom 96-well microtiter plates purchased from NUNC (South San Francisco, CA, USA). SPN lymphocytes were seeded in triplicate with calcein-AM labeled Yac-1 cells at various effector-to-target (E:T) ratios (from 100:1 to 12.5:1). Between 1 × 10^6^ cells to 1.25 × 10^5^ SPN cells and 1 × 10^4^ Yac-1 cells were mixed in each well. After incubation at 37 °C in 5% CO_2_ for 4 h and centrifugation, each supernatant was harvested and transferred into new plates. Samples were measured using a Spectra Gemini microplate spectrofluorimeter (excitation filter: 485 ± 9 nm; band-pass filter: 530 ± 9 nm) (Molecular Devices, Sunnyvale, CA, USA). Data were expressed as arbitrary fluorescent unit (AFU). The ratio of specific lysis was calculated according to the formula [(test release − spontaneous release)/(maximum release − spontaneous release)] × 100. Spontaneous release represents calcein-AM release from Yac-1 cells in medium alone, and maximum release is the calcein-AM release from Yac-1 cells lysed in medium plus 2% Triton X-100, each measured in at least six replicate wells.

### 4.13. ELISA

The concentrations of cytokine and antibodies in cell culture supernatant, plasma and ICs were measured using a commercially available enzyme-linked immunosorbent assay kit: OptEIA^TM^ mouse IL-12 (p40) ELISA was purchased from BD Pharmingen. Mouse IgG quantitation kit and mouse IgA quantitation kit were purchased from Bethyl Laboratories (Montgomery, TX, USA). Anti-OVA IgG in plasma and anti-OVA IgA in the ICs were measured as previously reported, with some modification [28]. Briefly, Maxisorb ELISA plates (Nunc) were coated with 20 μg/mL OVA (Seikagaku Corp., Tokyo, Japan) at room temperature, overnight. After washing and blocking, collected plasma or the ICs were added and incubated at room temperature for 2 h. HRP-conjugated anti-mouse IgG (Bethyl Laboratories) or HRP-conjugated anti-mouse IgA (Bethyl Laboratories) was used for detection. TMB was used as the substrate (e-Bioscience), and the reaction was stopped by the addition of 1M phosphoric acid. Absorbance was measured at 405 nm.

### 4.14. Statistics Analysis

All values are expressed as means ± SD. Statistical differences in the in vivo study between two groups were determined with the unpaired, two-tailed Student’s *t* test, (*p* < 0.05). Data from the in vitro study were analyzed using one-way ANOVA with Bonferroni post-hoc test. All statistical analyses were performed using the Excel-Toukei 2015 software program (Social Survey Research Information, Tokyo, Japan).

## Supplementary Materials

Supplementary materials can be found at www.mdpi.com/1422-0067/19/1/64/s1.
