# Supplementary material for: Modulation of Innate Immunity by lignin-Carbohydrate, a Novel TLR4 Ligand, Results in Augmentation of Mucosal IgA and Systemic IgG Production
Source: Int J Mol Sci. 2017 Dec 26;19(1):64. doi: 10.3390/ijms19010064 (PMC5796014; doi:10.3390/ijms19010064)
Supplement: Supplementary file 1 [file ijms-19-00064-s001.pdf]

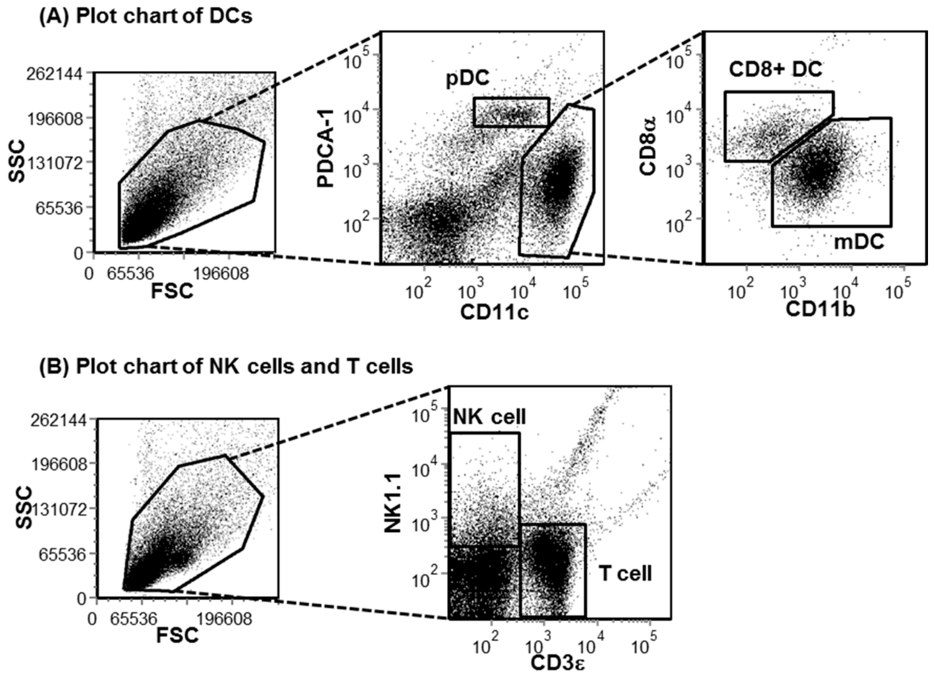

Figure S1. Representative FACS plot charts of DCs, NK cells and T cells.

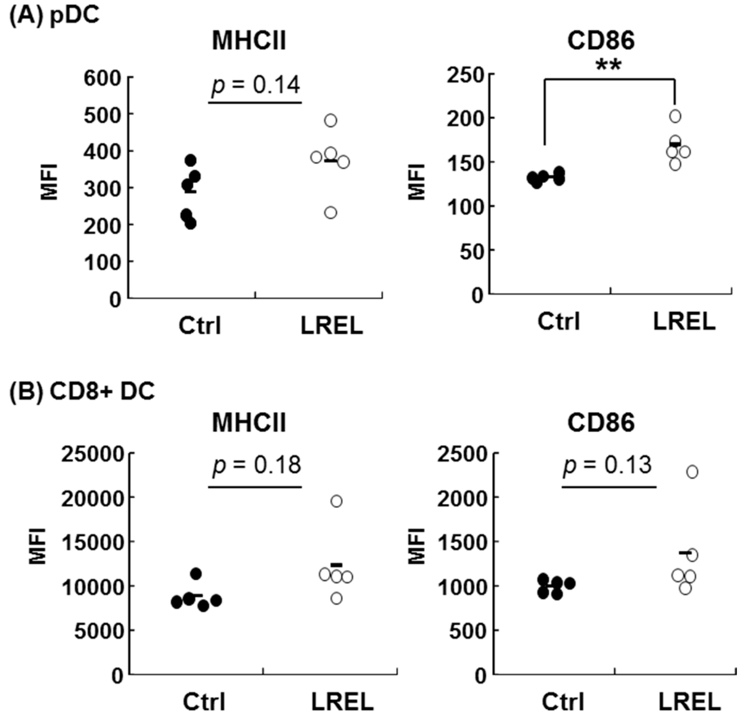

Figure S2. Comparison of MHCII and CD86 expression levels on SPN pDC and CD8+ DC. The expression levels of cell surface activation markers were analyzed by flow cytometry and evaluated as median fluorescence intensity (MFI). pDCs were defined as "CD11c^{int} PDCA-1^{-}"and CD8+ DCs were defined

as “CD11c^{+} CD11b^{-} CD8α^{+} PDCA-1^{-}“ in the low density cells of splenocytes. Data are shown as mean ± SD (*n* = 5). **, p < 0.01, compared to control (Student’s *t* test). Experimental conditions were same as Fig. 1.

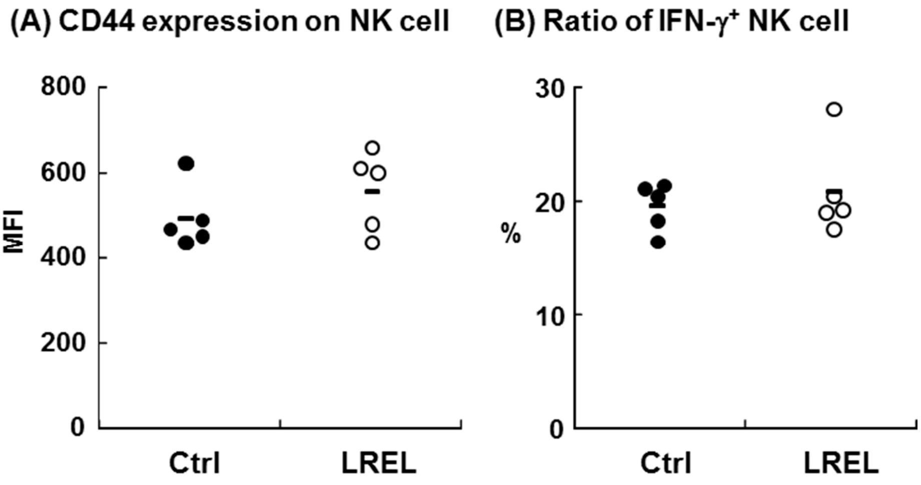

Figure S3. Comparison of NK cells activities in MLN. The activities of NK cells were evaluated by flow cytometry and NK cells were defined as “NK1.1^{+} CD3ε^{-}“. **(A)** CD44 expression level on MLN NK cells. The expression levels were evaluated as MFI. (B) Ratio of IFN-γ^{+} NK cells in MLN. Experimental conditions were same as Fig. 3.

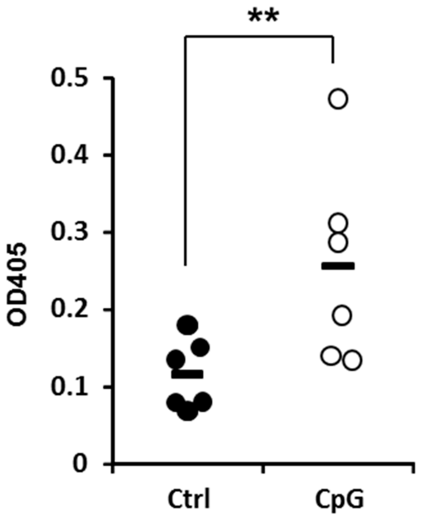

Figure S4. Examples for anti-OVA IgA measurement. In preliminary experiment, anti-OVA IgA concentration was measured in case of OVA administration, and found that there are no problems for the method of anti-OVA IgA measurement.
